# An Inclusive History of LGBTQ+ Aversion Therapy: Past Harms and Future Address in a UK Context

**DOI:** 10.1177/10892680241289904

**Published:** 2024-10-07

**Authors:** Kate Davison, Katherine Hubbard, Sarah Marks, Hel Spandler, Rebecca Wynter

**Affiliations:** 1 University of Edinburgh, Edinburgh, UK; 2 3660 University of Surrey, Guildford, UK; 3 4894 Birkbeck, University of London, London, UK; 4 6723 University of Central Lancashire, Preston, UK; 5 1724 University of Birmingham, Birmingham, UK

**Keywords:** aversion therapy, LGBTQ+ psychology, LGBTQ+ history, pathologisation, truth and reconciliation, conversion therapy

## Abstract

From the 1950s, aversion therapy gained an international foothold as a behaviourist means to alter what was then considered ‘deviant’ behaviour. Using primary research by psychologists, psychiatrists and other clinical figures published in professional journals, recently published personal testimonies by those who underwent such ‘treatment’, and drawing on the latest historical research, this article maps aversion therapy practices used to ‘treat’ LGBTQ+ people in the UK, mainly in the 1960s and 1970s. We outline our approach to this history and contextualise it by drawing attention to ongoing comparative issues of banning LGBT conversion therapy in the present. Next, we outline the emergence of aversion therapy internationally and identify historical ‘hotspot’ hospitals and universities in the UK, with the nation itself an international ‘hotspot’ for aversion. We then employ the case study of the 2022 report from the University of Birmingham, to demonstrate how such investigations of difficult pasts might be most effectively realised and highlight the potential for a ‘truth and reconciliation’ approach to this history. Finally, we call upon psy-organisations, university and research institutions, and other stakeholders to take this history seriously in effort to address past and ongoing harms enacted upon LGBTQ+ people.

## Introduction

Historically, sexualities and genders considered ‘non-normative’ have been actively pathologised and stigmatised within the psy-disciplines, medicine and wider society (Bayer, 1987; Minton, 2002). This pathologisation led to the development of various clinical interventions to try to change people’s sexuality or gender expression to align with dominant social norms and ideals – the assumption being that it was preferable for people to be heterosexual and identify with the sex they were assigned at birth, and the gender expression expected to be associated with it.

One of the most controversial and damaging ‘treatments’ was ‘aversion therapy’, a specific technique informed by behaviourist theory developed in the mid-20th century and practised in the UK primarily in the 1960s and 1970s. Behaviourism is most famously associated with Russian physiologist Ivan Pavlov and American psychologist B.F. Skinner; its primary theorisation is that behaviour can be altered by using conditioning techniques with stimuli (see Davison, 2021; Marks, 2015; Rutherford, 2009). LGBTQ+ aversion therapy applied such behaviourist principles and sought to alter someone’s sexual behaviour or their gender presentation to socially acceptable norms by utilising such conditioning. Precisely because aversion therapy was often applied with these explicit aims, it was one of the collection of practices now known as ‘conversion therapies’, a much broader term that includes all techniques, whether medical, religious, or otherwise, whose aim is to undermine a person’s self-determined sexual or gender identity. This term has undergone various iterations, which may (inadvertently or otherwise) serve to sanitise praxis, or else distance contemporary people and professions from a difficult past. In this article, we use quotes that employ other phrases, for example, ‘sexual orientation and gender identity or expression conversion practices’, but centre on the specific practice of aversion therapy.

During the period in which aversion-based ‘treatments’ were practised in the UK, being lesbian, gay, bisexual or trans, or any inflection of queer – the term used as a slur prior to its (at least partial) reclaiming from the 1970s – was not socially accepted, even within otherwise socially progressive or ‘alternative’ communities. Indeed, being LGBTQ+ was actively stigmatised, shunned, and medicalised, and in some cases explicitly criminalised. The internationally influential *Diagnostic Statistical Manual* (DSM) of the American Psychological Association, and the World Health Organization’s International Classification of Diseases (ICD), labelled ‘homosexuality’, ‘transsexuality’, and ‘transvestism’ mental disorders (see King et al., 2004; Minton, 2002). While some criticism was expressed within the psy-professions from the start, it was not until the arrival of the gay liberation movement in the late-1960s that these diagnoses and practices were effectively challenged (Bayer, 1987; Davison, in press; see also Hegarty, 2018; Spandler & Carr, 2022). In this context, it is worth noting that the majority of those who underwent such ‘treatments’ did so ‘voluntarily’, albeit often under substantial duress – in some instances from family members influenced by wider social prejudices or from the courts, especially in the case of men who had sex with men (Dickinson, 2015).

Not all psy-professionals advocated or utilised such methods. Some clinicians actively opposed them and eventually developed alternative approaches aimed at encouraging individuals to accept their sexuality or gender expression and reducing distress caused by social stigma (see Dickinson, 2015; Hubbard, 2021; Minton, 2002; Stapleton, 1975). Others, however, may have criticised aversion therapy’s focus on behaviour, but still practised other forms of conversion therapy, for example, via psychoanalysis or psychotherapy. It is crucial to recognise that none of the professional psychotherapeutic paradigms – whether behaviourist, psychoanalytic, or otherwise – were free of such attempts (Herzman & Newbigin, 2023).

It is also important to note how, while we might understand such histories as being related to LGBTQ+ people, these are recent identity constructions. We use these terms with careful consideration throughout – our aim here is not to apply terms inappropriately to those in the past but to recognise that this history is highly relevant to LGBTQ+ communities in the present. When we do use such terms we do in full awareness of the potential for anachronism and accept this as sometimes necessary to recognise LGBTQ+ narratives within this history. Likewise, we align ourselves with other affirmative histories who have taken a similar approach (e.g. Giffney, Sauer, Watt, 2011; Heyam, 2023; Hubbard, 2020). A critical history of aversion therapy is necessary to contextualise and understand past and present efforts to alter someone’s gender expression and/or sexuality. We refer to this as a queer and trans affirmative history and argue it remains necessary to the current political moment (see Kunzel, 2018).

Although sexuality and gender are distinct, they overlap and intersect in many complex ways (Kneale et al., 2019). Historically, concepts of sex, gender, and sexuality have often been conflated, both within and outside of medical and scientific disciplines. This conflation led Hubbard and Griffiths (2019) in their history of LGBTQ+ Psychology in Britain to argue that these histories are diverse, yet entangled, and deserve to be recognised as such. As such, we draw distinctions between those within LGBTQ+ communities whilst maintaining awareness of their intertwined histories. Drawing attention to ‘treatments’ such as aversion therapy, they emphasised that

It is important to be mindful that professionals often believed that their work would enhance the well-being of LGBTIQ people, even as this proved not to be the case at the time, and certainly not with historical hindsight. Understanding how and why these interventions were once normative but are no longer is part of the task of critical history. It also helps develop the capacity for critical reflection on current practices and highlights the need to develop ‘expertise’ that incorporates the lived experiences of LGBTIQ people (Hubbard & Griffiths, 2019, p. 942).

Although LGBTQ+ aversion therapy fell out of favour in the psy-professions after the mid-1970s, it did not disappear altogether. Jowett (2020) charted LGBTQIA-related research in British Psychological Society (BPS) from 1941 to 2017 and highlights this ideological shift from ‘treating’ homosexuality and to a lesser extent ‘transexuality’ until the late 1970s to focusing on prejudice from 1980 onwards. In most cases of aversion therapy specifically, practitioners ceased using and promoting it because of low clinical ‘success’ rates, after many careers had already benefited by being seen to employ ‘cutting edge’ techniques. The gay liberation movement was broadly successful in challenging the ‘medical model’, which led to the partial removal of ‘homosexuality’ from the DSM and ICD. However, this did not put an end to pathologisation (see Bartlett et al., 2001; Bayer, 1987; King, 2003; Minton, 2002), much as the partial decriminalisation of male homosexuality in 1967 in England and Wales did not put an end to police harassment of LGBTQ+ people (Jivani, 1997).

This reminds us that ‘progress’, as Hubbard and Griffiths (2019) have observed, although hard-won, is often precarious and restricted. Key examples of this in a UK context are the long-lasting anti-LGBTQ+ effects of Section 28 – which legally forbade what was termed the ‘promotion of homosexuality’ in UK schools between 1988 and 2003 – and the slow response by authorities to HIV/AIDS. Hubbard and Griffiths (2019) highlighted how changes that ‘appear positive can take longer than anticipated for their effects to be felt and this sometimes only impacts a select group of people’ (p. 951), often leaving more marginalised people on the sidelines. In the case of the DSM and ICD, gender non-conforming identities and certain forms of same-sex desire continued to be pathologised for several decades afterwards and some of these remain on the books today (see Bayer, 1987; Minton, 2002; Riggs et al., 2019).

Aversion therapy is not recorded as being currently practised in the UK and no official psychological organisations or institutions condone its use. Nonetheless, forms of conversion therapy remain. Although we distinguish between these, as aversion therapy is a specific psycho-behavioural technique, it remains within the family of conversion practices. The UK Government’s *Conversion Therapy: An Evidence Assessment and Qualitative Study* (2021) identified that efforts to change LGBTQ+ people’s sexuality tended to be run by faith groups, mental health professionals, or family members and often ‘take the form of talking therapies or spiritual guidance and intervention’ (Jowett et al., 2021; see also the commissioned report for Ireland, Keogh et al., 2023). Moreover, a proposed ban on LGBT conversion therapies in the UK, first brought to British parliament in 2018, has been repeatedly hindered by efforts to remove gender identity from the bill. If successful and the British parliament were to enact an amended version, it would make conversion therapy for lesbian, gay, and bisexual sexualities illegal, but practices targeting trans people based on gender identity would remain legal. In their open response to the consultation on banning conversion therapy, the BPS argued that there was a need to recognise the context in which such ‘therapies’ take place:

Conversion therapies are typically premised on the assumption that being heterosexual and/or cisgender is preferable to being LGBT+ and on the assumption that being LGBT+ is a disorder, defect, deficiency or addiction. It is important to recognise that conversion therapy in the UK takes place within a socio-cultural context in which LGBT+ identities have been historically stigmatised and in some sections of society continue to be so (see Bajwa, 2022).

A UK-based history of aversion therapy, as a facet of conversion therapy more broadly, is therefore very timely. A trans inclusive and affirmative history is especially vital given the steep rise of anti-trans sentiments in the UK over the past few years (Butler, 2024; Horbury & Yao, 2020; Pearce et al., 2020). This includes transphobia in mainstream media (Gupta, 2019; Montiel-McCann, 2023) and an increase of hate crime towards trans people in England and Wales (rising by 11% in one year from 2022 to 2023, Government Statistics, second Ed). Butler (2024) has contextualised anti-trans hostility within the UK within the global far Right conceptualisation of ‘gender ideology’ and comments that the UK appears to be especially inflamed in its political and social discussions about trans rights. They also note that the UK’s current legal procedures stand:

in defiance of a growing set of international norms, which maintain that a simple act of self-determination ought to suffice for changing one’s legal status, and that subjecting trans and genderqueer people to elaborate surveillance, inspection, diagnosis, and pathologisation is both unnecessary and harmful (p. 147).

The fact that a bill first proposed in 2018 has been repeatedly blocked from the order of debate in British parliament (unlike countries such as Argentina, Uruguay, Canada, New Zealand, and France) demonstrates the current state of public discourse around trans rights in the UK. However, the Labour Government, who came into power in July 2024, have promised to implement a ban on conversion therapy for both sexuality and gender identity.

This article charts the history of LGBTQ+ aversion therapy in the UK. It draws on the primary research published by psychologists, psychiatrists, and other clinical figures about their aversion praxis, more contemporary historical research, and the accounts published by those who underwent such ‘treatment’ to demonstrate how aversion therapy targeted people across the LGBTQ+ spectrum. Firstly, we briefly provide a historical overview of the emergence of aversion therapies internationally and then outline the use of aversion therapy in UK universities and hospitals. Secondly, we identify ‘hotspots’ where aversion therapies were used on LGBTQ+ people. Here, we highlight how people who might now identify as, or be recognised as, trans are evident in this history. Thirdly, we outline what is being done to recognise this history by institutions and organisations involved in such practices in the past, paying particular attention to the case of the University of Birmingham, an internationally renowned higher education facility in the English Midlands. Here, we consider the potential value of a ‘truth and reconciliation’ approach in acknowledging the harms caused by aversion therapy. Finally, we consider the legacies of aversion therapy in the present. In conclusion, we argue that to truly account for and reconcile this difficult history of aversion therapy at national and international levels, it is vital to take a queer and trans affirmative stance and consider how it has impacted people from all LGBTQ+ communities in the past as well as in the present.

## Aversion Therapy and Its Growth in British Hospitals and Universities

Aversion therapy emerged after the Second World War as one among several new clinical techniques grouped under the heading ‘behaviour therapy’ and became a global phenomenon during the second half of the 20th century (Alexander, 2023; Davison, 2021; see for example Freund & Srnec, 1953; Rachman, 1961). It was built upon theories of learning and conditioning developed in the early 20th century by Russian neurologists Vladimir Bekhterev and Ivan Pavlov and American psychologists Clark Hull, Edward Thorndike, John Watson, and B. F. Skinner. Behaviour therapists believed that conditioning techniques could be used to ‘treat’ a wide array of ‘neuroses’, which were considered to be a consequence of ‘unadaptive learned behaviour’ leading to conditions including phobias, social anxiety, or obsessive–compulsive disorder (Eysenck, 1960; Wolpe, 1954). People who presented with a diverse range of symptoms including writer’s cramp, stammers and tics, smoking, ‘obsessional’ ruminations or rituals, agoraphobia, eating disorders, and gambling were ‘treated’ with conditioning techniques (e.g. Liversedge & Sylvester, 1955; Thorpe et al., 1964). These techniques varied, but on the whole they aimed to encourage the positive adoption of normative behaviour. In the case of phobias, for example, conditioning treatment could involve graded exposure to the source of fear combined with relaxation, in a process known as systematic desensitisation (Lazarus & Rachman, 1957). These principles were later fused with cognitive therapy to produce what we today refer to as cognitive behavioural therapy or CBT, the most widespread talking treatment in the UK and in Global Mental Health settings (Marks, 2015). The British medical establishment was central to the development and international spread of behaviour therapy, and via the nation’s colonial vectors it became especially popular across the Commonwealth (Davison, 2021).

Behaviour therapy was promoted as a humane, empirically grounded, efficient alternative, because it targeted symptoms rather than causes, insofar as these symptoms manifested in human actions that were considered to be abnormal, pathological, criminal, or otherwise socially undesirable (e.g. Eysenck, 1960). A major driver in the development of aversion therapy was a dissatisfaction among some professionals with other psychological treatment paradigms, including hormonal and surgical methods, but especially psychoanalysis (Davison, 2021). In contrast to psychoanalysis, the underlying theory of aversion therapy was one of simple learning and unlearning, without attention to symbolism or the psychological power of early parental attachment relationships: if the symptom, or behaviour, could be unlearned, the ‘neurosis’ was considered to be cured. The goal was to ‘correct’ or ‘modify’ behaviours that were compulsive, uncontrollable, or learned by mistake (Davison, 2021). Aversion therapy attempted to do this by forging an association between these ‘undesirable’ behaviours and unpleasant bodily sensations (Marks, 2015).

LGBTQ+ aversion therapy typically took the form of presenting an individual with sexualised images, text, or objects – the ‘stimuli’ – and simultaneously giving them electric shocks or emetic drugs to make them sick (Davison, 2021; Dickinson, 2015). If the ‘undesirable’ behaviour they presented with was ‘homosexuality’, then the stimuli used were homoerotic. If they presented with ‘transvestite’ or ‘transsexual’ behaviours, then the stimuli centred on ‘cross-dressing’ (e.g. Barker et al., 1961). It was hoped that by overwriting the pleasurable feelings prompted by these stimuli with negative feelings, an ‘aversion’ to the associated desires, expressions, and practices could be produced. The level of active involvement by patients depended on the specific variation of the technique. Where images were used in combination with electric shocks, for example, the person would sometimes be able to control the slide projector themselves so they could actively avoid a shock. At other times, they discovered that this button on the projector had been disabled. This was known as the ‘anticipatory’ method, where waiting for a shock caused additional distress and was designed to prolong discomfort or provide the illusion of being in control of the process (see Birmingham Report, 2022; Dickinson, 2015; Spandler, 2020; Spandler & Carr, 2022). There were many variations, each method slightly tweaking the timing, sequence, which parts of the body the electrodes were attached to, adding or subtracting hormone injections, and so on, but they all reflected the same overall template (Davison, in press).

Aversion therapy was not exclusively developed to target LGBTQ+ people. One of its earliest main uses was in the ‘treatment’ of alcohol addiction, where patients would be instructed to take sips of alcohol after being injected with a nausea-inducing drug such as emetine (e.g. Franks, 1958). Yet behaviours relating to sexuality and gender – grouped crudely as ‘paraphilic’ – were of major interest (Davison, in press). We say crudely, because ‘paraphilias’ encompassed a wide range of non-normative feelings around sexuality and gender, whether harmful or not, that were not socially accepted. This meant that queerness, kink, and gender diversity were lumped together with violent and abusive behaviours. Same-sex desire, trans identity, and cross-dressing were placed side by side with paedophilia and exhibitionism (now acknowledged to be a form of sexual assault), and they were all equally considered to warrant psychological ‘treatment’. It was therefore common in the medical literature of the time for them to be discussed together, as variations of a single phenomenon, placing extra demands on historians for critical reading of the sources, as we discuss in detail below. While British behaviourists enthusiastically embraced aversion therapy for this full array of distinct gender and sexuality issues, LGBTQ+ people were a major focus (Davison, in press).

The wellspring of behaviour therapy in the UK was the Institute of Psychiatry at Maudsley Hospital in London, where many leading aversion therapy practitioners of the 1960s and 1970s trained there under the guidance of Hans Eysenck, Professor of Psychology from 1955 to 1983, who was a vehement critic of psychoanalysis, a proponent of the view that IQ might be determined by race, and a zealous advocate of behaviour therapy (Buchanan, 2010; Davison 2021; Marks, 2015). This included the psychologist Maurice Phillip Feldman, who trained at Maudsley before taking up a position at Crumpsall Hospital in Manchester (now re-named North Manchester General Hospital), where he and others conducted the largest and most widely published clinical experiments in the UK using aversion therapy on LGBTQ+ people (Birmingham Report, 2022). Prominent aversion therapists and psychiatrists Michael Gelder and John Bancroft also trained at Maudsley, where their experiments targeted LGBTQ+ people (Drucker, 2014; Marks, 2015). Gelder would later go on to author the *Oxford Textbook of Psychiatry* and Bancroft became the director of the Kinsey Institute, the world’s most prestigious centre for sexology research (King & Bartlett, 1999). Cyril Franks, another psychologist, worked at Maudsley in the 1950s, experimenting with conditioning and aversion techniques before emigrating to the US, where he became a founding member and the first president of the Association for the Advancement of Behavior Therapy (AABT) (Buchanan, 2010). These examples illustrate the significant international reach of the Maudsley and its trainees in the growth and spread of aversion therapy.

The Maudsley was also a place where psychiatrists and psychologists from outside the UK came to learn and exchange behaviourist principles and techniques. In the mid-late 1950s, Eysenck invited two South African emigres, the psychiatrist Joseph Wolpe and his PhD student Arnold Lazarus, a psychologist, to London to ‘aid in his fight against traditional psychotherapy’ (Cooper & Nicholas, 2012; Poppen, 1995). They would later follow Cyril Franks to the US to become the subsequent two AABT presidents. Wolpe and Lazarus were quickly joined in London by a third South African and collaborator, Stanley (‘Jack’) Rachman, who became Eysenck’s PhD student, as well as another South African psychiatrist Isaac Marks, who worked closely with Bancroft and Gelder at the Maudsley. Wolpe, Lazarus, Rachman, and Marks were all key players in the use of aversion therapy to treat sexual ‘deviations’. Further international visitors around this time included the Australian psychiatrist Neil McConaghy, who later became one of the most prolific practitioners internationally of homosexual aversion therapy, his best friend Sydney Lovibond, a psychologist who became a major promoter of behaviourism in Australia, and McConaghy’s role model, the Czechoslovakian psychiatrist Kurt Freund, who ran a globally pioneering clinical experiment with homosexual aversion therapy between 1950 and 1953 (Davison, 2021). This experiment so impressed Eysenck, that he personally translated Freund’s German-language report and published it in English (Davison, 2021; Eysenck, 1960; Freund, 1960).

While the Maudsley was the epicentre of British behaviourism, psychiatric hospitals and university clinics and research facilities across the UK were active sites for aversion therapy and its use on LGBTQ+ people. A rich source for mapping its reach are the professional medical journals, where we can see the authors’ institutional affiliations. Three of the most important journals were *Lancet*, the *British Journal of Psychiatry* and *Behaviour Research and Therapy*, established by Eysenck in 1963 (Eysenck, 1963).

Hospitals were the primary location of actual aversion therapy practices. ‘Hotspot’ hospitals in the UK included St George’s Hospital (Bancroft & Marks, 1968; Raymond, 1964) and Charing Cross Hospital Psychiatric Unit (Bancroft et al., 1966) in London, Banstead Hospital in Surrey (Barker et al., 1961; Thorpe et al., 1964), Barrow and Glenside Hospitals in Bristol (Cooper, 1963; James, 1978), Highcroft Hospital in Birmingham (Fookes, 1969), and Southern General Hospital in Glasgow (McGuire et al., 1964), to name just a few. The largest documented use of LGBTQ+ aversion therapy in Britain occurred at Crumpsall Hospital in Manchester, where Maudsley-trained psychologist Feldman (mentioned above) and psychiatrist Malcolm MacCulloch carried out extensive clinical studies spanning several years (see Jones, 2011). MacCulloch arrived at Crumpsall after graduating in medicine from the University of Manchester in 1960, to begin specialising as a psychiatrist. Feldman and MacCulloch forged a fast partnership that would see them research together intensively for the next decade. Their research attracted a generous donation specifically for the purpose of ‘curing’ homosexuality (Birmingham Report, 2022; Hubbard, 2020). Experimental clinical trials with aversion therapy at Crumpsall began in 1962 and the researchers began to publish their results from 1964.

Feldman and MacCulloch initially explored different forms of behaviour considered ‘deviant’ using people referred to, or found, at Crumpsall. Among their patients were people labelled as alcoholics, ‘transvestites’, and those exhibiting other ‘sexual aberrations’ (Feldman et al., 1968; MacCulloch et al., 1966). But – perhaps due to MacCulloch’s interest in what is now termed forensic psychiatry, and his mentor Dr. Northage de Ville Mather’s courtroom work (Birmingham Report, 2022) – their aversion therapy experiments swiftly began to focus on ‘homosexuality’ (Feldman, 1968; Feldman & MacCulloch, 1971). What that meant, however, lacked precision. Keen to solve the problem of low ‘success’ rates reported in the literature, Feldman and MacCulloch developed a specific variation of aversion therapy called ‘anticipatory avoidance’, which they claimed was more effective at reorienting sexual inclinations away from homosexual and towards heterosexual behaviour. In 1965, they reported their preliminary results of using this method on 19 people in Eysenck’s journal, *Behaviour Research & Therapy* (Feldman & MacCulloch, 1965). In June 1967, just as the Sexual Offences Bill to partially decriminalise male homosexual acts was being debated in the Houses of Parliament, Feldman and MacCulloch were reporting their first full trial results encompassing 43 people (according to them, 41 men and 2 women) in the esteemed *British Medical Journal* (Feldman & MacCulloch, 1964). By the time of their first monograph, *Homosexual Behaviour: Therapy and Assessment*, in 1971, the men were reporting on up to 77 people, ‘treated’ in two Manchester trials (Feldman & MacCulloch, 1971). The only other longitudinal studies in ‘homosexual’ aversion therapy to parallel Feldman and MacCulloch’s work were those led by Kurt Freund at Charles University in Prague from 1950 to 1957 encompassing 67 patients using the apomorphine method, and Neil McConaghy at the University of New South Wales and Prince Henry Hospital in Sydney from 1964 to 1981, who by 1973 had ‘treated’ ‘over 200’ male and ten female patients using various methods including both apomorphine and electric shocks (Davison, 2021; Davison, in press). The second biggest single study within the UK was carried out by John Bancroft, Marks, and Gelder at Maudsley, reporting on 40 patients (Bancroft & Marks, 1968). Most other published studies indicated much smaller cohorts of fewer than five or ten patients, or they were single case reports.

While the patient cohort numbers listed above appear low, it is safe to assume that they do not reveal the full extent of LGBTQ+ aversion therapy. We know, for example, that Feldman ran a cottage industry of ‘treating’ people who approached him (correspondence with Wynter, 2022) whose cases were not counted among the published clinical trial data or medical records (Spandler, 2020; Spandler & Carr, 2022). These people found their way to him privately, or through organisations such as the Albany Trust, or via referrals from a local GP (correspondence with Wynter, 2022; London School of Economics Archive, HCA/ALBANY TRUST/8/48; Hunte, 2020). This was likely true of other published aversion therapists too, not to mention unknown numbers of psychiatrists and psychologists who copied the techniques described in the literature without publishing their results. Inspired by Feldman and MacCulloch’s research, additional local clinical trials at Hollymoor Hospital – a psychiatric site close to the University of Birmingham – offered further opportunities for clinicians to use aversion therapy on a range of people, identities and behaviours, well into the 1970s (see James, 1978; James et al., 1972; James, Orwin, & Turner, 1977; James, Carter, & Orwin, 1977; Turner, Pielmaier, et al., 1974; Turner, James, & Orwin, 1974).

As the above examples illustrate, universities provided important institutional backing for aversion therapy research. Following their clinical trials and data gathering at Crumpsall Hospital, Feldman and MacCulloch both took positions at the University of Birmingham, where they continued to process and publish their findings (Birmingham Report, 2022). Other sites of higher education in the UK likewise supported LGBTQ+ aversion therapy by providing its practitioners with status, legitimacy, research infrastructure, and bona fides. John Bancroft and Michael Gelder, for example, after years of clinical experimentation at Maudsley, were appointed at the University of Oxford in 1969 to establish its Department of Psychiatry, where Bancroft continued to publish on LGBTQ+ aversion therapy (Bancroft, 1974), before moving to the Centre for Reproductive Biology at the University of Edinburgh in 1976. Ralph McGuire had also taken up a position at Edinburgh in the Department of Psychiatry in 1971, after many years at Leeds University where he published findings from his aversion therapy trials at Glasgow, celebrated in a 2012 obituary as ‘seminal’, career-defining work (McGuire et al., 1964; Peck, 2012). Affiliation to universities also facilitated access to research funding via grants. Where the costs of aversion therapy research were not contained in-house by hospitals and university clinics, it was sometimes provided by outside support, including from the publicly funded Medical Research Council (e.g. Bancroft & Marks, 1968). This demonstrates the dual complicity of hospitals and universities in this history.

## LGBTQ+ Aversion Therapy – A Full Spectrum

There has been a common misconception that aversion therapy exclusively targeted gay men. This view is not supported by the literature, nor by personal testimonies, which make clear that men and women who showed any same-sex desire, trans people, and queer people more generally were all subjected to aversion therapy (e.g. Collier, 2023; Evans, 2019; Hunte, 2020). Based on the published literature alone, it is reasonable to assert that while men with sexual desire towards other men were most likely to undergo the ‘treatment’ (Bartlett et al., 2009; King et al., 2004; Smith et al., 2004), the next largest LGBTQ+ target group included people who ‘cross-dressed’ and those who may now be understood as trans, followed by cisgender lesbian or bisexual women (Carr & Spandler, 2019; Spandler & Carr, 2022).

Contemporary research about trans people’s experiences of aversion therapy is still emerging, yet there is plenty of primary evidence in the psychiatric, psychological, and medical literature of its use in the ‘treatment’ of gender non-conformity. The word ‘transvestism’ may confuse readers today, but at that time, it was a broad term incorporating a range of expressions of gender identity and was not limited to ‘cross-dressing’. Along with other variations such as ‘transsexualism’, ‘transsexuality’ and ‘transvestitism’, and sometimes even ‘fetishism’ or ‘fetishist’, these terms were often used interchangeably throughout the 1960s (see Blakemore et al., 1963a, 1963b; Clark, 1963; Glynn & Harper, 1964). Sometimes they were distinguished from one another, but not reliably. For this reason, it is crucial that historians, psychologists, psychiatrists, and other researchers read the literature carefully and with a sense of flexibility. Even individuals who were categorised as ‘homosexuals’ back then may not have been ‘gay’ as we understand that term today.

In fact, the earliest published report we have found of aversion therapy being used on non-normative sexuality or gender in the UK was a ‘paper [that] describes the treatment of a male transvestite’ ‘treated’ at Banstead Hospital in Surrey, and reported in the *Lancet* in March 1961 (Barker et al., 1961; see; Lavin et al., 1961, p. 347). Lavin et al. (1961) distinguished between ‘transvestite’ and ‘transsexual’ categories and placed their 22-year-old ‘patient’ clearly in the former. We approach this historical work from a trans affirmative perspective, which in methodological terms means that we intentionally create space for the possibility of trans experiences and identities when interpreting source materials. There is of course no way to know how the subjects of aversion therapy would identify now (beyond survivor accounts). Yet, what is possible to deduce is how various forms of gender non-conformity were ‘treated’ with aversion therapy and how these are relevant to LGBTQ+ histories now. The ‘treatment’ this person underwent in this study was described in elaborate detail in the published reports (Lavin et al., 1961). In preparation for ‘treatment’, the doctors photographed them in women’s clothing and made an audio recording of their own voice describing themselves getting dressed, piece by piece. They then made them look and listen to these media while injecting apomorphine every 2 hours, for 6 days. Only fluids were supplied for the first 2 days, after which they were given a ‘light diet’. During the ‘treatment’, the person subjected to it felt a ‘deep sense of humiliation’ and, based on the published report, suffered a rapid physical breakdown which included ‘rigors and a temperature of 99°’, high blood pressure, impaired coordination, being unable to hold a conversation, and the abandonment of the final four sessions (Lavin et al., 1961).

Numerous single case reports like this can be found throughout the 1960s (cf. Thorpe et al., 1964), but there is also ample evidence of larger cohorts and longer uses of aversion therapy on people who might today have identified as trans (see for example Blakemore et al., 1963a, 1963b). In 1964, McGuire, Carlisle, and Young at Southern General Hospital in Glasgow reported to have ‘treated’ five ‘transvestists’ using an electrical aversion therapy method (McGuire et al., 1964). In the same year, three Maudsley researchers reported results of a study of 19 people being ‘treated’ for ‘transvestism’, 13 of whom underwent aversion therapy (Morgenstern et al., 1964). Further research on this theme was carried out by Gelder and Marks at the Maudsley, sometimes at the behest of the patients’ families: in these cases, the apomorphine method was discarded and replaced with electrical or ‘faradic’ shocks, because the former was considered to cause too much discomfort (Gelder & Marks, 1967). Together with Bancroft, they then carried out an experiment with ‘electric aversion in 40 male patients including 14 transvestites and transsexuals’ (Bancroft & Marks, 1968). Between 1956 and 1969, Michael Raymond at Fairdene and Netherne Hospitals in Surrey ‘treated’ 13 people for ‘transvestism’ (Raymond, 1965, 1969), while B. H. Fookes at Highcroft Hospital in Birmingham included five ‘fetishist-transvestists’ among 27 patients in his aversion therapy trial (Fookes, 1969). In a review of the literature in 1966, Feldman counted around 30–35 cases in the UK where aversion therapy was used to ‘treat’ people who ‘cross-dressed’ in the UK, alongside around 83 cases of male homosexuality (Feldman, 1966), and from the rest of the published literature these relative proportions seem to have remained steady throughout the 1960s and 1970s. Here, too, we must keep in mind the likely ‘cottage industry’ of private ‘treatment’ that might have been operating beyond these studies, meaning that the number people ‘treated’ with aversion therapy in the UK, including trans people, was probably much higher than the published reports suggest.

Feldman and MacCulloch’s work is one of the clearest indicators of the profound muddle in the application and understanding of terminology. Indeed, they created much wider confusion through their taxonomies of what they termed ‘primary’ and ‘secondary’ homosexuality (Birmingham Report, 2022). According to them, so-called primary homosexuals posed a greater therapeutic challenge because they had never fantasised about or enacted heterosexual behaviour. By contrast, secondary homosexuals, considered from their life histories to have shown at least some heterosexual tendencies at some point – some of whom might have described themselves as bisexual in today’s language – were framed as more likely to elicit ‘successful’ outcomes. This already questionable division became even more dubious with their assertion that ‘we consider trans-sexuals as homosexuals by definition’ (Feldman & MacCulloch, 1971, p. 177). According to them, the association of primary homosexuality

with sex-inappropriate childhood behaviour raises the question of the relationship between primary homosexuality, trans-sexualism … and transvestism, occurring in homosexuals. In our view, these three types of sexual deviation are probably closely related … The problem is to explain why only a minority of primary homosexuals (who are then termed trans-sexuals) not only display sex inappropriate behaviour, but in addition wish to change their bodily appearance so that morphology and behaviour will be consistent (Feldman & MacCulloch, 1971, p. 177).

The conceptual and terminological slippage here strongly suggests that trans women were caught in the researchers’ dragnet and categorised as ‘male homosexuals’. Indeed, as Spandler and Carr have noted, ‘at least some of the people recorded as being treated as (male) transvestites [in Feldman and MacCulloch’s publications] may actually have been (trans) women (or, at least, might have been if the wider culture had enabled this)’ (Spandler & Carr, 2022, p. 222).

There is therefore a vast array of evidence from aversion researchers themselves that demonstrates how aversion therapies were thought to be suitable for a range of people who were considered to be outside of cisgender and heterosexual norms. Furthermore, it is not only that psychiatrists and psychologists in the 1960s and 1970s did not always make clear distinctions between sexual orientation, gender identity, and actual practices – some of their patients probably did not either and may have traversed between the various yet limited identity borders that were available to them at the time. Despite, or perhaps precisely *because* of these blurred edges, the evidence safely puts to rest any false claims that people who would today identify or be perceived as lesbian, trans, bisexual, and queer were not targeted for aversion therapy. It also shows how necessary it is to carry out thorough historical, psychological, or psychiatric research that is sensitive to definitional changes as well as institutional and political contexts, and that listens to a variety of voices without prefiguring what they might be telling us.

Such sensitive historical work has been in development. Almost 25 years ago, King et al. (2004) reviewed British psychiatry and paid particular attention to ‘treatments’ offered for homosexuality. Several years later, they collaborated with Glenn Smith and published two oral history articles on the ‘treatments’ of homosexuality from the 1950s. One focused on the oral histories of patients (Smith et al., 2004) and the other on the oral histories of the professionals conducting such ‘treatment’ (King et al., 2004). Together, these papers emphasised the long-term damaging impact the, often behavioural, ‘treatments’ had on individuals and supplied a warning that decisions around pathology are related to social and political assumptions. Dickinson (2015) in *Curing Queers* examined oral histories of gay men and trans women who received aversion therapy, and the nurses who administered such therapy, in Britain in the middle of the 20th century (also see Dickinson et al., 2012). Importantly, this work demonstrated how sometimes the mental health nurses supporting such ‘treatment’ were themselves queer but understood their work as helping others who were not happy with their sexualities. The oral histories also revealed some supportive connections made between nurses and patients. Davison (2021) mapped the transnational use and circulation of LGBTQ+ aversion therapy in two critical waves between 1950 and 1975 in Czechoslovakia, Germany, and the British Commonwealth (also see Davison, in press). In an effort to pay closer attention to women who were ‘treated’ with aversion therapy, Spandler and Carr (2020; 2022) identified at least ten women ‘treated’ for same-sex desire in the UK in the 1960s. They used diverse sources for their historical account which further highlighted the extent to which coercion was involved in apparently ‘voluntary’ ‘treatments’.

Alongside this historical analysis, survivor testimonies have also been published (e.g. Collier, 2023; Evans, 2019; Gavins, 2018; Hunte, 2020; Jones, 2022). One of the women featured in Spandler and Carr (2022) was Pauline Collier, who was also one of the two women included in a 1967 paper by MacCulloch and Feldman, ‘Aversion Therapy in the Management of 43 Homosexuals’ in the *British Medical Journal* (MacCulloch & Feldman, 1967). She has recently written about her experience in *The Psychologist* (Collier, 2023), offering a rare and valuable first-person account. Likewise, Carolyn Mercer has spoken about her experiences of aversion therapy as a trans woman. She was ‘treated’ with electrical aversion therapy ‘by NHS doctors … at a hospital in Blackburn’ in 1964 (Evans, 2019; Loffhagen, 2022). Jeremy Gavins, too, published a memoir in 2018 detailing how his experience of aversion therapy as an 18-year-old schoolboy in Bradford in 1972 negatively shaped his life in deep and lasting ways (Gavins, 2018; Strudwick, 2017b). Such personal testimony has had a substantial effect in recognising the true impact of aversion therapy on LGBTQ+ individuals. This is important, not only methodologically but can also help other institutions who may have been complicit in the harms caused by aversion therapy to come to terms with their ethical responsibilities.

## The Birmingham University Report and Subsequent Apologies

In late 2020, a man called ‘Chris’ approached a BBC journalist to talk about his experience of being ‘treated’ with aversion therapy at the University of Birmingham in the mid-1970s. The BBC published the story (Hunte, 2020). The initial response by the University was to distance itself from the claims. However, it was eventually persuaded by some of its own academic staff to fund an investigation into its historical involvement with sexual reorientation practices. The results were published in a report entitled ‘“Conversion Therapy” and the University of Birmingham, *c*.1966–1983’, which was written by one of the current authors (Wynter). Accompanied by a public apology from the Vice Chancellor, who condemned conversion therapy as ‘unethical, degrading, and harmful’, the report was launched in June 2022, and published on the University’s website (Birmingham Report, 2022). The University of Birmingham is the first such institution in the UK (and to the best of our knowledge, in the world), to acknowledge its connection with aversion therapy and issue a public apology.

The report focused on the work of two aversion therapists – Feldman and MacCulloch – who were recruited to academic positions at the University of Birmingham in 1966 and 1967, respectively. The report found that although a majority of the work Feldman and MacCulloch published during their time at Birmingham was based on clinical experiments they had undertaken in Manchester prior to their appointment, there were at least two sets of aversion therapy apparatus on premises used by the University, one located in the city and one on campus. It was also revealed that one of the people to receive aversion therapy from MacCulloch during his time at Birmingham was a 12-year-old boy who was ‘treated’ in 1969/70 for ‘exhibitionism’ towards older women (Birmingham Report, 2022). This shows, along with the Report’s analysis of their terminological slippage, that the harms caused by ‘treatment’ methods aimed primarily at LGBTQ+ people also encompassed other individuals who would not be categorised in this way, but who undoubtedly suffered unjustifiable pain.

The report demonstrates the extent of hospital and university staff time expended on aversion therapy–related activities, and how organisational involvement did not stop at the University or at Hollymoor Hospital, but included the Birmingham Regional Hospital Board, Marston Green Maternity and John Connolly Hospitals in Birmingham, Aston University, and the Lucas Group Research Centre at Solihull near Birmingham (Birmingham Report, 2022). Blaming two men alone ignores the network that supported the practice of aversion therapy, a network that was likely repeated at each of the ‘hotspots’ for aversion praxis in the UK and elsewhere in the world. New historical research is now revealing the involvement of these institutions, who are beginning to recognise how the power they held allowed these practices to occur (Birmingham Report, 2022; Davison, in press; Spandler & Carr, 2022).

As more historical research into aversion therapy becomes available, not to mention information concerning other medical, psychological, and psychiatric conversion techniques, it is becoming clearer how the power held by hospitals and universities not only allowed these practices to occur but in most cases even won them prestige and recognition. Many aversion therapy practitioners built and boosted their careers in this field of clinical experimentation. They often received support in kind or funds to develop their work, received positive reviews, gained promotions, and secured professional awards. It is time to begin to acknowledge, understand, and learn from these mistakes. For owning its history and issuing an apology over 50 years later, the University of Birmingham should be commended. Yet the case of Birmingham is also illustrative of a severe shortcoming in institutional engagement: it was not until ‘Chris’ approached a journalist, and that journalist decided to publish his account, that the University was pushed to investigate its own responsibilities for the harm it supported and facilitated. This pattern around scandals dictating how complaints are heard and find redress is, unfortunately, well established, as described by sociologists Butler and Drakeford (2003). They argue repeated scandals and serial malpractice has meant psychiatric treatment in the UK has become highly regulated. Likewise, historians Reinarz and Wynter (2015) have argued that patterns of complaint in medicine (including the psy-disciplines) go back centuries. Now that Birmingham has led by example, we urge other university and hospital institutions to take greater initiative.

Survivors of LGBTQ+ aversion therapy have also attempted to approach psychological, psychiatric, and medical authorities with their testimonies in the hope it will result in official acknowledgement of the harms they suffered (e.g. Collier, 2023; Evans, 2019; Hunte, 2020). Yet apologies from these authorities have been a long time coming and are rarely unprompted. In 2017, the President of the Royal College of Psychiatry issued an apology for psychiatry’s complicity in the oppression of gay people by treating homosexuality as a mental disorder (Strudwick, 2017a). Once again, this was prompted by gay survivor Jeremy Gavins who came forward to tell his story to *Buzzfeed* journalists (Strudwick, 2017b).

Following on from the Royal College of Psychiatrists’ apology, and almost contemporaneous with the publication of the Birmingham Report, the American Association of Behavioural and Cognitive Therapies published an ‘Apology for Behaviour Therapy’s Contribution to the Development and Practice of Sexual Orientation and Gender Identity and Expression Change Efforts’ in 2022. The statement denounced the use of ‘conversion therapies’ and stated that the organisation

deeply regrets behavior therapists’ role in the creation, study, and use of these practices, and recognizes and accepts responsibility for the ways in which both our actions and inactions have harmed SGM [sexual and gender minority] people. ABCT recognizes it is time for us to document our history and legacy and say that we are truly sorry (ABCT, 2022).

The statement drew considerable criticism from some of the organisation’s own members for not going far enough, and for its concluding emphasis on the need for clinicians to ‘educate’ themselves about the profession’s past failures – instead, they argued, senior members of the ABCT who had been involved in the practice of aversion therapies should have been named (Flaherty, 2022). A further criticism, which the ABCT President Laura Seligman has taken up, targets the continued inclusion of research articles relating to aversion and conversion therapies in online back issues of major journals, including the ABCT’s own *Behavior Therapist* journal, with calls for retraction (Seligman, n.d). It is worth quoting from Seligman’s letter to highlight the legal and ethical challenges that remain in relation to the debate about article retractions:

Dr. Carolyn Becker (who serves as the Board liaison to the Membership Issues Committees, including the Sexual and Gender Minority SIG) and I also consulted with several attorneys on all aspects of this important issue. We found out that some publishers bring retracted articles out from behind the paywall to make the retraction notice more available. Additionally, we learned that retractions would have no impact on the admissibility of study findings in court. Thus, we continue to investigate the retraction issue and have turned our focus more broadly to additional ways that we could have meaningful impact here.

To date, this has included investigating several options, including disclaimers that would make clear the harms that result from the use of SOGIECEs [sexual orientation and gender identity or expression conversion practices], the development of a white paper and/or clinical guidelines that could be used by attorneys and others who are also trying to limit harms to the LGBTQ+ community, and working with our Publications Committee to consider a special issue in one of our journals to document (a) the consequences of SOGIECEs and (b) the limitations of the research that SOGIECEs proponents continue to use (Seligman, n.d).

Retractions certainly have the potential to discredit the historical clinical and scientific claims that may yet still be used to support conversion therapies and ongoing harms to LGBTQ+ people. This is one of the many areas where active and sustained engagement with stakeholders from across professional and scientific organisations and LGBTQ+ communities could open discussion on how harms could be mitigated in future.

More recently, the Cummings Center for the History of Psychology in Akron, Ohio, had to similarly contend with an issue of historical legacy and the practises of conversion therapy. The Center is named after Drs Nicholas and Dorothy Cummings because of their substantial financial support, yet Nicholas Cummings was a strong advocate for conversion therapy practices if it was requested by a client (see Faye, nd). It was not until 2015, aged 91 that his perspective appeared to have changed. An extensive report of Cummings and his work on conversion therapy is provided (Winston, 2023, with an open letter by the Cummings family to ‘our partners within the LGBTQIA+ community’) alongside the statement made by the Center on its position. Here, they also encourage engagement with the American Psychological Association’s Resolution on Sexual Orientation Change Efforts (2021).

There is no doubt that the personal testimonies of survivors have had a powerful cumulative effect, galvanising others to come forward. Pauline Collier was inspired to publish her account of experiencing aversion therapy in *The Psychologist* (Collier, 2023) in the wake of ‘Chris’ approaching the BBC and the University of Birmingham’s 2022 report. Chris’s story, the Report’s assertion that without survivor testimonies knowledge of what happened would be incomplete, and the research by Spandler and Carr (2020), fostered Collier’s hopes of finally receiving recognition for the suffering she endured at the hands of Feldman and MacCulloch at Crumpsall. Although the Hospital has not issued an apology and it has so far proved impossible to locate any medical records or archives detailing events during this time, there have been some positive developments. In response to Collier’s account, Jim Orford, a psychologist who worked with Feldman and MacCulloch at Manchester when he was new to the field, wrote a heartfelt and ‘unreserved’ apology for his involvement in these ‘treatments’ (Orford, 2023). In the wake of the Birmingham Report, the University of Manchester has also made a firm commitment to investigate its role in the historical development and practice of aversion therapy (private meetings and correspondence with Wynter and Spandler) and has appointed a researcher to investigate.

Writing on behalf of the British Psychological Society (BPS), Adam Jowett, Chair of the Equality, Diversity and Inclusion Board, and Dr. Debra Malpass, Director of Knowledge and Insight, have reflected on how ‘Pauline’s experience of how psychologically and emotionally exhausting it is to suppress one’s LGBT+ identity resonates with the accounts of those who have experienced conversion therapy more recently’, citing a recent government report, and stating that the BPS ‘unequivocally opposes conversion therapy practices’ (Jowett, 2020). The BPS established a ‘Challenging Histories’ group that reports to the Ethics Committee. This is an interdisciplinary group that includes BPS members, psychiatrists, and historians reviewing some of the darker aspects of psychology’s past (https://www.bps.org.uk/ethics-committee) (British Psychological Society, 2023; Jowett & Malpass, 2023).

Some psychiatric survivors have advocated for a ‘truth and reconciliation’ type approach to harm caused by psychiatric and psychological interventions (Spandler & McKeown, 2017; Wallcraft & Shulkes, 2012). Sometimes described as ‘restorative practices to harm’ or simply ‘restorative practices’, these start by acknowledging that mistakes were made. It also involves carefully and truthfully documenting these mistakes, not only to help prevent future wrongdoing but to begin recognising the immediate and ongoing effects of the medical mistreatment of minorities, including, but not limited to, gender and sexual minorities (Spandler & Carr, 2022). Truth and reconciliation approaches in South Africa at the end of Apartheid exposed the use of aversion therapy to ‘treat’ homosexuality for those in the military until at least 1978 (see Reddy et al., 2013). Aubrey Levin, a devout supporter of Apartheid, was the head psychiatrist in ‘The Aversion Program’ which operated in the South African Defence Force. Reports about the torturous use of aversion therapy, hard labour, and chemical castration under the leadership of Levin emerged (Van Zyl et al., 1999) and he was later convicted of sexual assaulting patients in Canada in 2013. The instance of Levin again points to South African figures and to praxis in former British Dominions.

Further applications of truth and reconciliation approaches for harm caused by psychological intervention are emerging. For example, in May 2022, the Department of Health in Victoria, Australia, commissioned advice to the Minister for Mental Health on how their government could formally acknowledge historic harms in the mental health system. This resulted in the *Not Before Time* report, which explicitly advocated a restorative justice approach to psychiatric harm (Katterl, 2023). Such restorative processes would benefit from rigorous and ethically driven historical (and contemporary) research to surface the harms experienced. Therefore, we see the work outlined here as being part of a long-term strategy to achieve social justice for those people affected by these practices, but also to achieve a more encompassing and proactive commitment to affirmative and health-focused care going forward.

The application of truth and reconciliation style approaches to LGBTQ+ aversion therapy carries with it the same limitations that have been identified in relation to such approaches more broadly (see Avruch, 2010; Rose, 2015). Spandler and McKeown (2017) explored such challenges directly in relation to mental health services and argued that truth and reconciliation processes must be carefully adapted to context. For LGBTQ+ aversion therapy specifically, one aspect that limits the potential for reconciliation is that too often institutional acknowledgements have only surfaced in response to individual claims made by survivors from LGBTQ+ communities. There are several problems with this. Firstly, it is crucial for institutions to take the initiative and not to wait for individuals to come forward, expecting them to relive experiences that may have been extremely painful then and may continue to cause harm. This suggests that an institution has no case to answer until proven otherwise by the testimony of a living survivor. Moreover, it suggests that the acknowledgement of harms caused by aversion therapy is only relevant in relation to specific individuals and not to whole communities of LGBTQ+ people, whose social stigmatisation, rejection, and oppression was bolstered by such practices and their continued valence in the marginalisation of sexual and gender minorities as pathological. Secondly, there are members of LGBTQ+ communities who may not be in a position to come forward with personal accounts, precisely because of misconceptions about who was targeted for aversion therapy and because of ongoing stigma and prejudice.

Thirdly, there is a danger in addressing institutional apologies exclusively and directly to people who today are identified as ‘lesbian’, ‘gay’, ‘bisexual’, or ‘trans’, because it sets up a false and ahistorical moral judgement between ‘deserving’ and ‘undeserving’ recipients of aversion therapy. Not only is it often impossible to accurately identify those people who did, or would now, identify as LGBTQ+ in publications, it is also impossible to reliably distinguish who was being referred to when terms like ‘fetishists’ or ‘sexual deviants’ were used (see Clark, 1963). Limiting the acknowledgement of harms only to these present-day identity groups suggests that aversion therapy was otherwise justifiable; as the example of the 12-year-old boy in the Birmingham Report shows, this is simply too short-sighted. Institutional acknowledgements for harmful practices must be fulsome and must not create a false hierarchy among survivors.

Fourthly, a ‘truth and reconciliation’ approach can be limited if it assumes that ‘reconciliation’ can be achieved, and if it demands forgiveness on the part of those who experienced these harms. Such an expectation cannot and must not be a starting point for institutional acknowledgements of harm (Spandler & McKeown, 2017). It may also anticipate survivor testimonies, reflecting a uniform narrative of trauma, when experiences are actually diverse. For example, whilst most aversion therapy recipients experienced the intervention as negative, and many suffered extensive and long-lasting trauma, for others the experience was not one of indelible harm and they were soon able to laugh at the crude theories of their doctors (Davison, in press). Finally, restorative justice demands more than an acknowledgement of past harms. It also requires positive action as part of a future-oriented process of collaborative and inclusive work to prevent similar harms from being perpetrated in the future.

## Conclusion

We have charted the history of LGBTQ+ aversion therapy in the UK and synthesised it with the current UK context and contemporary literature. In doing so, we have brought together disparate materials, accentuated the extent of the diversity of aversion therapy in relation to gender and sexuality, and argued for proactive restitution. We have demonstrated that wider LGBTQ+ communities were considered suitable for such ‘treatment’, and people who would now identify as, or be recognised as, trans are especially evident in this history. This is important to emphasise given the ongoing clinical pathologisation of trans and gender non-conforming people.

Throughout the 1960s and 1970s, when aversion therapy was at its height, terminology often conflated or collapsed groups we now consider more distinct across gender identity and sexuality lines. It remains essential to consider such terms carefully, but also to recognise the significance this history has for LGBTQ+ individuals in the past and present. By doing so, we have been able to reveal the extent to which aversion therapy in the UK impacted and targeted wider LGBTQ+ communities. Such a history is very timely for two reasons: (1) institutions and organisations are finally beginning to reconcile with their own histories as survivor testimonies emerge and (2) the prevalence of wider discussions of banning conversion therapy.

While some readers might feel reassured that the LGBTQ+ aversion therapy practices described in this article are a thing of the past, its legacies and the ethos behind it remain very much in the present. The UK Government LGBTQ+ Survey (2017) found that 5% of LGBTQ+ people had been offered ‘conversion’ or ‘reparative’ therapies in Britain and 2% had undergone conversion therapies (sometimes called ‘reparative’, ‘explorative’, ‘ex-gay’, or ‘sexual reorientation’ therapies). Notably, 13% of trans respondents had been offered or undergone conversion therapy, compared to 7% of cisgender lesbian, gay, or bisexual respondents.

Broadly speaking, although it is no longer considered clinically acceptable to try to reorient someone’s sexuality to the social norm of heterosexuality (at least within UK public health settings), it is seen as acceptable in other clinical settings to attempt to reorient someone’s gender to the social norm of the sex they were assigned at birth (O’Malley et al., 2023). Indeed, we have witnessed a growth of specific interventions such as (the misleadingly and euphemistically entitled) ‘gender exploratory therapy’ which actively steers clients away from being trans (Ashley, 2023).

The British Psychological Society has recently been involved in challenging these practices. They co-signed an updated ‘Memorandum of Understanding on Conversion Therapy’ in 2017 which widened the definition of conversion therapy to include gender identity (see Moon, 2021, for an explanation and history of this Memorandum). However, in early 2024 the UK Council for Psychotherapy withdrew its signature from the updated ‘Memorandum’ and its membership of the Coalition Against Conversion Therapy on the grounds of not wanting to oppose conversion therapy for trans young people. This decision faced significant criticism and at the time of writing, the matter had not yet been resolved (Memorandum of Understanding on Conversion Therapy. Version 2, 2022).

In light of this history and of the current, fast-moving context of the UK, we implore organisations and institutions who were involved with LGBTQ+ aversion therapy to document and account for these past actions and attempt to reconcile with the communities it has hurt. Genuine engagement is crucial with relevant stakeholders and individuals making the effort to understand the extent to which these histories remain in the present. Such work is critical, not only in terms of taking stock of what has happened but also to ensure that the mistakes of the past are learned from and not repeated.
